# Biosynthetic Mechanisms of Secondary Metabolites Promoted by the Interaction Between Endophytes and Plant Hosts

**DOI:** 10.3389/fmicb.2022.928967

**Published:** 2022-07-11

**Authors:** Zhaogao Li, Weie Wen, Ming Qin, Yuqi He, Delin Xu, Lin Li

**Affiliations:** ^1^Department of Cell Biology, Zunyi Medical University, Zunyi, China; ^2^Department of Immunology, Zunyi Medical University, Zunyi, China; ^3^Engineering Research Center of Key Technology Development for Gui Zhou Provincial Dendrobium Nobile Industry, Zunyi Medical University, Zunyi, China

**Keywords:** endophytes, interactive relationships, growth and development, secondary metabolites, biosynthesis mechanism

## Abstract

Endophytes is a kind of microorganism resource with great potential medicinal value. The interactions between endophytes and host not only promote the growth and development of each other but also drive the biosynthesis of many new medicinal active substances. In this review, we summarized recent reports related to the interactions between endophytes and hosts, mainly regarding the research progress of endophytes affecting the growth and development of host plants, physiological stress and the synthesis of new compounds. Then, we also discussed the positive effects of multiomics analysis on the interactions between endophytes and their hosts, as well as the application and development prospects of metabolites synthesized by symbiotic interactions. This review may provide a reference for the further development and utilization of endophytes and the study of their interactions with their hosts.

## Introduction

Endophytes are special “microorganism” with much value. They can parasitize different parts of living plants but do not obviously cause symptoms of external infection in host plants or symbiosis and coevolution with their host (Chen et al., 2022). As an important cooperative “partner” of plant growth and development, endophytes greatly impact on host physiological metabolism, which helps or stimulates host plants against various stresses (Koskimäki et al., 2022). More importantly, during this long-term association, the endophytes and their host form an interactive relationship of coevolution and mutually beneficial symbiosis. The genetics and metabolism of endophytes ameliorate and supplement the material metabolic pathway and related gene expression of their hosts (Mei et al., 2019). Conversely, the host also creates a unique community structure and gene characteristics of endophytes (Oukala et al., 2021; Li et al., 2022).

Exploring the application of endophytes has become a hot topic with the gradual understanding of the interaction between endophytes and hosts. Plants were first thought of as individual organisms that produced metabolites by themselves to completely support their growth and development. This cannot explain the phenomenon in which plants growing in the wild are “better,” such as in medicinal characters, than those under artificial cultivation; artificial cultivation of medicinal plants loses the original effect, etc. It was not until the discovery of endophytes that their interactive relationship was gradually revealed, especially after the application of omics technology at the cellular and molecular levels in which the study of the interactive relationship between the two progressed rapidly. In recent years, researchers have been trying to explore the evolutionary relationship between host and endophytes, and trace the reciprocity mechanism of this specific evolutionary relationship, in order to provide theoretical basis for the commercial development of endophytes. At present, some endophytic bacteria and their active products have been successfully used in commercial production and obtained great benefits in the preparation of new drugs and agricultural protection (Singh and Gaur, 2017; Mishra et al., 2018b; Sahu et al., 2021). It is of great economic value to reveal the interactive mechanisms between plants and endophytes to explore and release the potential for the industrial development of endophytes.

To provide a reference for revealing the complex interaction between endophytes and their hosts, this review first discusses the interaction between endophytes and host plants and then the effects of endophytes and their secondary metabolites on plant growth and development. The application of endophyte resources instead of is also discussed.

## Interaction Between Endophytes and Host Plants

### Plant Microecology Under Endophyte Invasion

Many plant biological functions are dependent on endophytes, and each plant is actually a complex microecosm (Compant et al., 2021). A broad consensus has been reached that endophytes and their host plants have had a coevolutionary history for millions of years. The interaction between them not only maintains ecological stability but also promotes the growth and development of both partners (Pérez-Alonso et al., 2020). With the improvement and application of cell and molecular science, omics and even “macro-omics,” the study of the interactions between endophytes and hosts has made great progress, and related theories, such as “mosaic theory,” “acquired immune system,” “equilibrium confrontation,” and “exogenous chemical excitation reaction,” have been proposed (Cui et al., 2017). In recent years, many studies on the interactions between endophytes and hosts have shown that the “balanced antagonism” theory is more accurate in explaining the relationship between endophytes and hosts. The core of the theory is that the “confrontation” between microorganism and plants is different from that of general pathogenic bacteria, and its essence is the special balance between the endophytic virulence factor and the plant defense and immune system; when the virulence factor is too strong, the plant will become sick. When defense stress is too strong, microorganisms are killed (Schulz et al., 1998). Actually, the balance of interaction between endophytes and hosts is far from a simple “confrontation” between virulence factors and the defense system, and its regulatory mechanisms are far more complex and precise than maintaining the balance between virulence factors and the defense system (Khaiwa et al., 2021).

**FIGURE 1:**
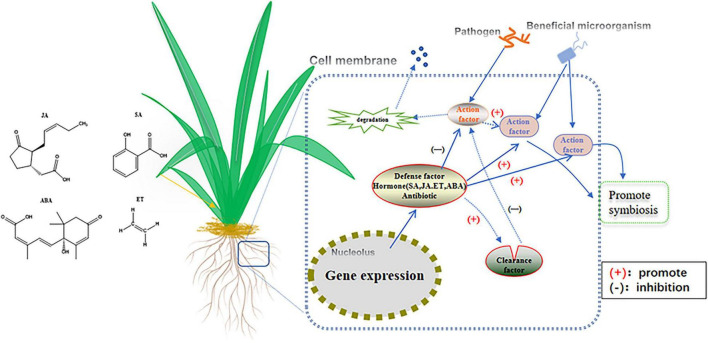
Signal response mechanisms for promoting (+) or inhibiting (–) plant – microbial interactions. Exogenous microorganisms release action factors to infest plants, which respond with gene expression and release metabolites such as antibiotics and hormones (SA, JA, GA, ET, and ABA) to interfere with the infestation to protect themself.

Further studies showed that when microbes invade the host plant tissue, the recognition of the plant self-defense system will begin the crosstalk of signal molecules to identify endophytes (Figure 1). For example, the medicinal plant *Camptotheca acuminata* kills invading microorganisms by producing camptothecin, which competitively inhibits the activity of the microbial topoisomerase I complex (Khaiwa et al., 2021). *Fusarium solani*, an endophytic fungus in *Camptotheca acuminata*, uses special amino acid residues to bind the relatively active domain of camptothecin to escape the attack of the host, while the other endophytes avoid the invasion risk by encoding a unique topoisomerase. Therefore, the colonization of endophytes is not easy (Sirikantaramas et al., 2009; Kusari et al., 2011, 2012a). Robinson’s results showed that the rhizosphere-dominant bacterium *Bacillus mycoides* could not colonize wheat in an aseptic system without competitive bacteria (Robinson et al., 2016). Further studies found that when competitive bacteria exist, wheat secretes special root exudates, which are rich in nutrients and can induce and promote *Bacillus mycoides* symbiosis (Robinson et al., 2016). In addition, Wang successfully colonized *rhizobia* in the roots of legumes by applying exogenous metabolites of flavonoids to realize the symbiosis of endophytes (Wang et al., 2021). Therefore, the formation of the interaction between endophytes and hosts is far from a simple combination of heredity and material between them but is accompanied by the overall cooperation of the “micro niche” formed by the internal and external environment of their hosts.

### Dilemmas in Interaction Mechanisms Study

Interactive mechanisms between endophytes and hosts are the material basis for the formation of interactions. Previous work has mainly explored the interactions between endophytes and their hosts from superficial aspects, such as morphological observations and chemical composition analysis. However, this does not fully explain the pathways of host growth, development and metabolism under the action of endophytes. At present, although the action mechanisms of some endophytes have been described, it is still difficult to accurately reveal the interactive mechanisms. The dilemmas are mainly manifested in three aspects: a large number of microorganisms react with plants at the same time, endophytes have signal interference, and symbiosis is difficult to simulate.

At present, it is very important to find and understand the growth mechanism of host endophytes. Studies suggest that endophytes affect plants mainly in two aspects. On the one hand, endophytes induced systemic resistance (ISR) production in the host. ISR differs from traditional system acquired resistance (SAR) in that its phenotype is similar to that of pathogen induced SAR, both of which can induce broad-spectrum resistance to pathogens in plants (Pieterse et al., 2014; Pozo et al., 2015). In essence, ISR differs from other induced-resistance mechanisms in that the host usually has primic defense associated with the jasmonic acid (JA) pathway under symbiotic association, and can initiate faster, stronger, and more durable defense expression under adverse conditions (Martinez-Medina et al., 2016; Teixeira et al., 2019). However, the mechanism of “priming” state is still unclear, and it is speculated that transcription factors and related signals play an important role in it (Bentham et al., 2020; Mengistu, 2020). On the other hand, endophytes can effectively regulate defense-related signaling pathways in the host to achieve self-colonization and thus establish symbiotic links with host plants. After colonization, endophytes invasion plants, are bound to across the plant immune system highly complex in the first line of defense, also known as microbial associated molecular patterns (MAMP), a process that involves many molecules [such as elongation factors Tu (*EF-Tu*), peptidoglycan (*PG*), superoxide dismutase (*SOD*), β-chitosan bacteria cold shock protein (*RNP1motif*), chitin, etc.] and the specificity of the host cell surface receptor molecules PRR identification (Newman et al., 2013). Under current conditions, although it is known that symbiosis with endophytes endows hosts with additional defense mechanisms, little is known about this aspect (Eid et al., 2021; Roy et al., 2021). In fact, plant immune system activation and mechanical changes are the result of ongoing molecular dialogue with endophytes, a common example of which is the JA pathway discussed above (Gutjahr, 2014; Pozo et al., 2015; Bastias et al., 2017). After plant infection with endophytes, the salicylic acid (SA) pathway is usually inhibited, the JA precursor level is enhanced, and then the JA reaction gene is upregulated (Wasternack and Hause, 2013). However, different from the SA pathway, the JA pathway is not well understood (Zhang M. J. et al., 2021). Existing studies believe that the increase of JA pathway seems to be related to the development and function of mycorrhizal symbiosis, which may be understood and verified in future studies (Siciliano et al., 2007).

### Application of Omics to Elucidate the Interactions Between Endophytes and Hosts

Endophytes are important biological resources. It is important to fully understand the interaction between endophytes and hosts to fully exploit the value of endophytes. Although research on endophytes has made great progress, they are still unknown in regard to many aspects of research value. At present, the rational use of scientific methods to fully understand the interaction between endophytes and hosts is helpful for providing more choices and possibilities in the study of the conversion value of endophytes. Modern high-throughput genomic technology provides a technical basis for exploring the potential value of endophytes. An in-depth analysis of endophytes from the aspects of sequencing, taxonomic classification, phylogeny and biological evolution has greatly promoted enthusiasm for endophyte research (Selosse et al., 2022). Genome-wide analysis of endophytes directly reflects the colonization preference and genetic characteristics of endophytes on different hosts. This greatly promotes the identification of related genes, such as host growth and development mechanisms, antibiotic production, insertion elements, endophytic secretory system, surface attachment and other metabolic processes (Subudhi et al., 2018). Moreover, genome-wide analysis has also been applied to explain the survival and evolution of endophytes in hosts (Singh et al., 2021). For example, whole genome sequencing analysis revealed the potential of endophytic fungus *P. indica* as a plant probiotic preparation. Genome-wide analysis of the endophytic bacterium *Pantoea ananatis* revealed the existence of genes encoding hydrolase, n-acyl high serine lactone synthase, superadhesion factor and fusylic acid resistance protein. This shows its great biological potential for commercial production (Wu et al., 2020). At present, the whole genomes of some endophytes have been sequenced, and the number is increasing (Table 1). This can not only intuitively reveal the changes in gene expression and genetic characteristics of plants under interactive relationships but can also serve as a systematic model for the study of interactions between endophytes and hosts.

**TABLE 1:** List of selected endophytes genome sequenced in last 5 years (2018–2022).

Microbial classification	Microbial endophyte	Sources	Functions	References
Endophytic actinomycetes	*Nocardia*	Various plants	Significantly modulates antibiotic and gene expression associated with plant growth-promoting compounds.	Nouioui et al., 2022
	*Streptomyces*	Various plants	Promote plant growth performance, including IAA and aminocyclopropane-1-carboxylate (ACC) deaminase production.	Worsley et al., 2020
Endophytic fungi	*Sporisorium, Ceratocystis, Fusarium*	*Saccharum officinarum*	Encodes genes associated with ethylene that regulate phosphate metabolism and produce IAA. Genes encoding hydrolases and oxidoreductases are involved in biofilm formation and the metabolism of those secondary metabolites associated with it.	Challacombe et al., 2019
	*Ascomycota phylum*	Various plants	Interacts with host plants by secreting various proteins that promote symbiotic associations.	Baroncelli et al., 2020
	*Paraphaeosphaeria*	Various plants	—	Fn et al., 2021
Endophytic bacteria	*Pantoea agglomerans* ANP	*Medicago sativa* L.	Helps to relieve the stress of host plants under drought and salinity stress, and participates in the dissolution of phosphate and glucose dehydrogenase.	Hameed et al., 2020
	*Burkholderia* sp. LS-044	*Oryza sativa*	Involves hydrolysis of chitin, regulation of gene expression for the preparation of aromatic compounds, and metabolism of aromatic compounds.	Guo et al., 2020
	*Enterobacter roggenkampii* ED5	*Saccharum officinarum*	In plant growth, it promotes biological control and stress tolerance, and assists plants in nitrogen fixation.	Ulrich et al., 2021
	*Stenotrophomonas* sp.	Various plants	Related to plant colonization, growth promotion and stress protection.	Flores et al., 2018
	*Klebsiella variicola* KvMx2	*Saccharum officinarum*	Promote plant nitrogen fixation process, regulate virulence stress.	Babalola et al., 2021
	*Bacillus cereus*	*Helianthus annuus* L.	Enhance protein-coding gene expression in various metabolic pathways.	Singh P. et al., 2021
	*Pantoea Ananatis* NN08200	*Saccharum officinarum*	It can promote the biosynthesis of plant synthesis of IAA and promote the growth of sugarcane.	Zeng et al., 2020
	*Bacillus licheniformis* GL174	*Vitis vinifera* L.	Helps grape plants cope with pathogen attacks and reduces the use of chemicals in vineyards.	Nigris et al., 2018
	*Pseudomonas viridiflava*	Various plants	As a pathogen, it can not only cause disease but also defend against biological invasion and reduce the abundance of host microorganisms. It also plays a role in disease resistance.	Lipps and Samac, 2022
	*Cronobacter* sp. JZ38	*Arabidopsis thaliana*	Increasing tolerance of plants to salt stress plays a role in plant growth promotion and antagonistic activity against pathogenic microorganisms.	Eida et al., 2020
	*Bacillus halotolerans* Hil4	Various plants	By secreting metabolic substances, preventing and controlling plant mildew.	Thomloudi et al., 2021
	*Roseomonas hellenica* sp.	*Alkanna tinctoria*	—	Rat et al., 2021b
	Cal.l.30	*Calendula officinalis*	Secretes lipopeptides which are secondary metabolites with anti – microbial activity.	Tsalgatidou et al., 2022
	*Delftia* sp. BR1R-2 and *Arthrobacter* sp. BR2S-6	Various plants	Enhanced expression of pathogen-induced plant defense-related genes (PR-1, PR-5 and PDF1.2)	Kurokawa et al., 2021
	*Rhizobia*	*Phaseolus vulgaris*	Involved in amino acid and carbohydrate transport and metabolic material enrichment, cofactor biosynthesis.	Aguilar et al., 2018
	*Bacillus endophyticus*	—	Reduce the virulence of the environment to which the plant itself is exposed.	Lekota et al., 2018
	*Leclercia adecarboxylata*	*Zea mays* L.	IAA is produced *in vitro* to generate auxin and promote plant growth.	Snak et al., 2021

In addition, based on the commonly used single omics technology, omics has been further developed into “macro omics,” which provides more powerful technical support for the study of the interactions between hosts and endophytes. However, the comprehensive application of polyrecombinationics in the field of endophytes research can often yield more comprehensive results. In addition, we should fully consider the complex “network” system of interactions between endophytes and hosts. Then, using omics technology to analyze the composition differences between individuals, and even the interactions between multiple individuals, in order to form a new model to reveal the nature of interactions between endophytes and hosts (Lin, 2015). In recent years, many researchers have carried out studies on the interaction between endophytes and hosts by using joint omics, but there is still a huge gap in the understanding of the interaction between endophytes and hosts. In the next stage, there is still a long way to go to realize the perfect combination of information technology and data and to study the interactions between endophytes and hosts in a real sense.

## Studies on the Secondary Induction Role of Endophytes in Interactions With Hosts

### Endophytes Promote Host Growth and Development

**FIGURE 2:**
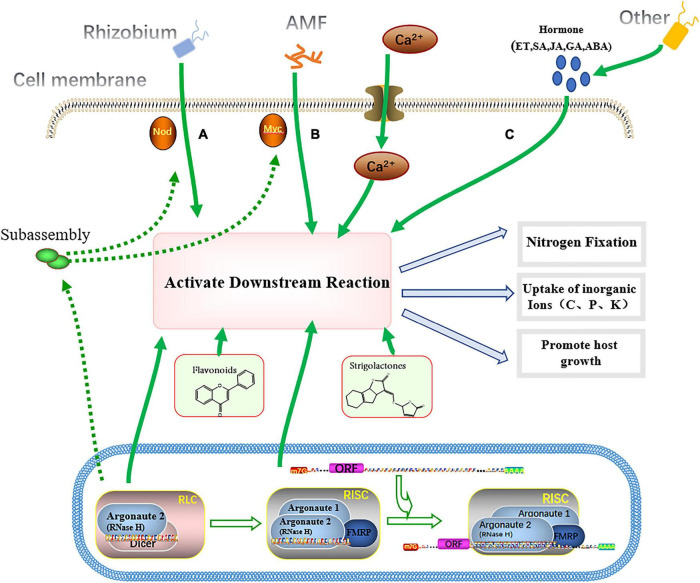
Endophytes affect host growth. Endophytes release factors that act on host cells, which express RNA-induced silencing complex (*RISC*) genes and silence specific genes, thereby regulating the invasion process of endophytes. **(A)** Under the action of Nod factor, rhizobia formed root nodules and fixed N_2_. **(B)** AMF form clumped mycorrhizae (AM) under the action of Myc factors, which promote host uptake of inorganic ions (C, P, N, etc.) and regulate the root environment. **(C)** Some endophytes are able to produce hormones (SA, JA, GA, ET, ABA, etc.) that promote host growth.

Endophytes can affect host growth and development by producing metabolites (Figure 2). Studies have shown that some endophytes can promote host growth by regulating hormones such as SA, JA, abscisic acid (ABA), endothelin (ET), and gibberellins (GA). For example, endophytes can reduce host ethylene levels by regulating 1-aminocyclopropane-1-carboxylate (ACC) deaminase activity. At the same time, the ability to synthesize plant hormone indole-3-acetic acid (IAA) is utilized to promote host growth and repair host activity during toxic damage (Glick and Stearns, 2011). Besides, some endophytes affect host growth through their own metabolism. For example, nitrogen-fixing endophytes in many hosts, such as soybeans and corn, can reduce free N_2_ from the atmosphere to NH_4_^+^ for host uptake and promote host growth (Jin, 2014; Guzmán et al., 2022). Some endophytes isolated from soybean root nodules promote the absorption of P by their hosts by dissolving P minerals (Varga et al., 2020). In addition, N, P, C and other elements were also stronger in soybean plants infected with endophytic arbuscular mycorrhizal fungi (AMF) than in uninfected soybean plants. This is mainly due to the rapid formation of special arbuscular mycorrhizal structures in soybean root AMF (Khaekhum et al., 2021).

### Endophytes Increase Host Stress Tolerance

Most endophytes enhance host tolerance to stress to some extent, while some plants may not survive due to lack of endophytes (Wu et al., 2021). The improvement effect of endophytes on their hosts is not only in the regulation of abiotic stress, such as tolerance to temperature, drought, salinity and metal ion stress, but also in the regulation of biological stress, such as resistance to diseases and insect pests and herbivorous organisms. Therefore, endophytes not only show a rich source of stress tolerance to their hosts but also show a wide range of effects.

#### Increase Stress Tolerance to Abiotic Stresses

**FIGURE 3:**
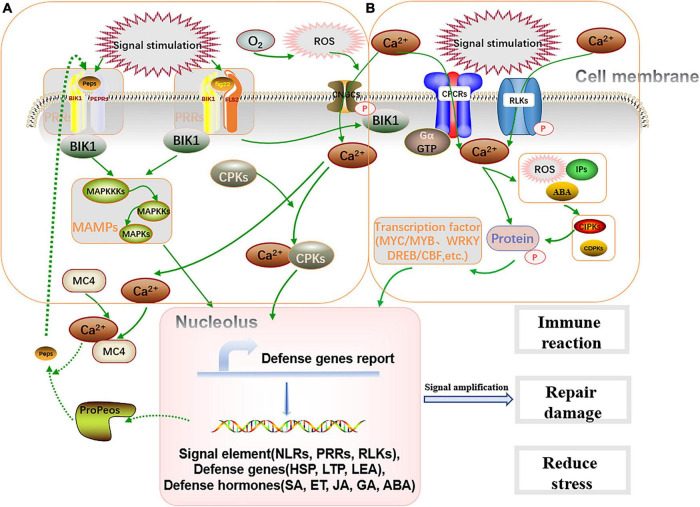
Endophytes regulate the tolerance of plants to stress. **(A)** Under stress environment, endophytes can produce exogenous signals and quickly activate plant *PRRs* membrane receptors. On the one hand, they activate the downstream MAPKs level connection On the other hand, they regulate the intracellular Ca^2+^ concentration. **(B)** Under the action of exogenous signal of endophytes, membrane receptors such as *CPCRs* and *RLKs* are activated to induce downstream reaction. Cascade signaling is transmitted in the nucleus to promote the expression of relevant response genes, so as to improve the ability of host in immune response, repair damage and reduce stress.

Endophytes can effectively regulate the stress tolerance of hosts to abiotic stresses (Table 2). Studies have shown that the diversity of endophytic communities or changes in metabolism have a significant impact on host adaptability to the environment (Li et al., 2022). Some endophytes activate membrane receptor molecular mechanisms and signaling pathways, such as G-protein-coupled receptors (*GPCRs*), receptor-like kinases (*RLKs*), ion channels, and histidine kinases, when the host is exposed to salinity, temperature, drought, and heavy metal toxicity. Change the concentration of Ca^2+^ in the cytoplasm and produce corresponding signaling molecules such as reactive oxygen species (ROS), ABA and inositol phosphates (IPs). Further kinase responses activate various downstream transcription factors, such as *MYC/MYB*, *WRKY*, *DREB/CBF*, *AREB/ABF*, *bHLH*, *NAC*, and *bZIP* (Sirikantaramas et al., 2009). Activated transcription factors lead to the activation of different stress-responsive genes, including the expression of lipid-transfer proteins (*LTPs*), heat shock proteins (*HSPs*), late embryogenesis abundant protein (*LEA*), antioxidant response element (*ARE*) and osmotic proteins, through reciprocal transformation of protein phosphorylation and dephosphorylation. Some endophytes activate pattern recognition receptor (PRR) membrane receptors under the influence of exogenous signals, amplify secondary signals through cascade reactions, and then activate the downstream MAPK signaling pathway, resulting in *NLRs*, *TFs*, *HSFA*2, *RLKs* and other related gene expression. Through the above series of biochemical reactions, endophytes can regulate host permeability to reduce or even offset the effects of stress and can finally achieve the purpose of repairing damage (Wang et al., 2020; Figure 3). Baek et al. (2020) found that salt stress-related genes were expressed in the shoots and roots of rice seedlings after infecting them with endophytic bacterium *Bacillus oryzicola* YC7007. AMF, an endophytic fungus isolated from chickpeas, can help the host to relieve drought stress (Hashem et al., 2019). In addition, under salt stress, some endophytic fungi can secrete exopolysaccharide to change soil structure, regulate soil material composition and change host permeability, so as to relieve stress (Kidd et al., 2021).

**TABLE 2:** Effect of endophytes on host abiotic stress tolerance.

Endophytic strains	Sources	Changes in matter	Functions	References
*Fusarium, Alternaria*	*Glycine max (Linn.) Merr.*	Triterpenoids, phenols and polysaccharides increased	Resistant to acid and alkali and other abiotic stress.	Xiao et al., 2021
*Bacillus cereus* SA1 *Rhizobium*	*Glycine max (Linn.) Merr.*	Increased SA, ascorbate peroxidase, superoxide dismutase, and glutathione	Resistance to high temperature.	Khan et al., 2020
*Burkholderia cepacia* J62, *Microbacterium* JYC17	*Brassica napus* L.	The contents of non-enzymatic antioxidants such as Ascorbic Acid(ASA) and Glutathione(GSH) were up-regulated	It inhibited heavy metal stress and increased the growth, antioxidant activity of copper absorption and microflora structure of *BRassica napus l*.	Ren et al., 2019
*Bacillus subtilis* (BERA 71)	*Cicer arietinum Linn.*	Increased levels of reactive oxygen species and lipid peroxidation	Under salt stress, increased chlorophyll synthesis in AMF treated plants was obvious, which lead to significant enhancement in the net photosynthetic efficiency.	Hashem et al., 2019
*Piriformospora indica*	*Artemisia carvifolia*	Flavonoids, ammoniated protein, superoxide dismutase and peroxidase activase were significantly increased	It can resist arsenic stress and improve the transcription level of genes and signal molecules in the biosynthesis pathway of isoprenodiol, terpene and flavonoids.	Rahman et al., 2020
*Sinorhizobium* *meliloti* CCNWSX0020	*Medicago lupulina*	—	It can resist nickel and cobalt stress and promote the growth of alfalfa in nickel and cobalt contaminated soil.	Li et al., 2018
*Bacillus* spp. *Arthrobacter* sp.	*Piper nigrum* L.	Pro accumulation	The growth of pepper under drought stress was promoted by Pro accumulation and ACC deaminase activity.	Saikia et al., 2018
*Bacillus megaterium* H3	*Oryza sativa* L.	—	It can resist arsenic stress and improve the ability of resisting bacterial invasion.	Cheng et al., 2020
*Bacillus* BM18-2	*Pennisetum purpureum*	Plant chlorophyll increases	It can resist cadmium stress, improve plant growth and repair soil health.	Kamal et al., 2021
*Aspergillus flavus* CHS1	*Chenopodium album*	Dissolution of phosphate, production of IAA and GA	Resistance to salt stress, promote chlorophyll, root length and other different plant growth characteristics.	Lubna et al., 2018
*Pseudomonas*	*Arabidopsis thaliana*	The contents of antioxidant enzymes and proline increased	Alleviate salt stress and repair plant growth conditions.	Fan et al., 2020
*P. indica*	*Nicotiana tabacum* L.	Peroxidase activity and glutathione content increased	Reduce the phytotoxicity of cadmium and enhance the activity of antioxidant enzymes.	Su et al., 2021
*Piriformospora indica*	*Arabidopsis thaliana*	The content of Pro, ascorbic acid and ABA increased, and the transcription level of related genes increased	Resistance to low temperature stress, improve the survival vitality of Arabidopsis thaliana under low temperature.	Jiang et al., 2020
*Bacillus subtilis* NUU4, *Rhizobium* ciceriic53	*Cicer arietinum*	—	Resistance to salt stress.	Lastochkina, 2019
*Meyerozyma caribbica*	*Zea mays* L.	IAA, phenols and flavonoids increased	Salt stress resistance, significantly increase root and stem length, plant fresh and dry weight, promote growth.	Jan et al., 2019
*Bacillus subtilis*	*Glycyrrhiza uralensis Fisch*	Flavonoid, polysaccharide and glycyrrhizic acid content increased	It can resist drought stress, improve the expression of *HMGR*, *SQS* and β -galactose glycyrase as the key enzymes of glycyrrhizic acid synthesis, and promote the accumulation of glycyrrhizic acid.	Zx et al., 2019

#### Increasing Stress Tolerance to Biotic Stress

Endophytes can enhance host resistance to biological stresses such as diseases and insect pests in a variety of ways (Table 3). On the one hand, some endophytes can avoid the competition of pathogenic microorganisms by producing lipopeptides, chitinases, pyrrolidine, glucanase, and other antibiotic metabolites (Mishra et al., 2018a). Baek et al. (2020) isolated the endophytic bacterium *Bacillus oryzicola* YC7007 from the rhizosphere of rice (*Oryza sativa*), which can induce systemic resistance and antibiotics, inhibit rice bacterial diseases and promote rice growth and development. The endophytic bacterium *Bacillus subtilis* EDR4 isolated from wheat competitively inhibited the growth of pathogens *Fusarium oxysporum* by producing the antifungal protein E2 (Liu et al., 2010). *Bacillus triticum* JC-K3, an endophytic bacterium isolated from wheat, can regulate local IAA concentration and then regulate the absorption of inorganic ions to promote the growth of host, thus alleviating the biological diseases under salt stress (Ji et al., 2021). On the other hand, endophytes can colonize hosts in a manner similar to the invasion of pathogens, which creates niche competition with pathogens (Ahmed et al., 2020). Therefore, plants can recruit endophytes and reduce the possibility of colonization of their own pathogens by giving priority to occupying the niche. Researchers isolated the endophytic bacterium *Stenotrophomonas* from *Pistacia chinensis*, which can preferentially occupy the niche by producing iron carriers, thus reducing the colonization of pathogenic bacteria and further relieving the iron stress environment of *Pistacia chinensis* (Etminani and Harighi, 2018). In addition, endophytes can also help hosts to cope with insect feeding. The endophytic alkaloid metabolites and neurotoxins may lead to disordered behavior, hindered growth and development, and even death of insects (Song et al., 2020; Tooker and Giron, 2020; Grabka et al., 2022). For example, *Bacillus* in sugarcane can induce the abnormal development of giant borers, thus reducing diseases and insect pests (Rocha et al., 2021).

**TABLE 3:** Effect of endophytes on host biotic stress tolerance.

Endophytic strains	Sources	Changes in matter	Functions	References
*Neotyphodium lolii*	*Lolium perenne*	—	Reduces aphid damage while reducing insect feeding.	Agriculture et al., 2018
*Bacillus subtilis* (BERA 71)	*Cicer arietinum Linn.*	Increased lipopeptides.	Promote plant growth and antifungal.	Abd-Allah et al., 2018
*Bacillus velezensis* JC-K3	*Triticum aestivum* L.	Produce IAA.	IAA increased, root biota increased, stem and leaf abundance decreased, and promoted inorganic ion uptake.	Ji et al., 2021
*Bacillus, Pseudomonas*, *Stenotrophomonas*, *Pantoea*, and *Serratia*	*Pistacia atlantica*	Siderophores, proteases, Hydrocyanic acid.	Competitive niche competition reduced the success rate of pseudomonas infection.	Etminani and Harighi, 2018
*Bacillus subtilis*	*Mangifera indica*	—	Bactericide (Bacillus subtilis) and increase the number of fruits.	Feygenberg et al., 2021
*Bacillus* sp.	*Triticum aestivum* L.	Chitinase, β- Glucanase, cellulase, lipase, and protease.	Regulate the up regulation of pathogenic related genes and inhibit the growth of pathogenic bacteria.	Diabankana et al., 2021
*Bacillus subtilis* EDR4	*Triticum aestivum* L.	Antifungal protein E2	Inhibiting the growth of pathogenic fungus *Fusarium oxysporum.*	Liu et al., 2010
*Bacillus* *subtilis strain* 1-L-29	*Camellia oleifera*	—	Anti-fungal infection.	Xu et al., 2020
*Bacillus subtilis* DZSY21	*Lycopersicon esculentum*	Hydrolase, IAA.	Anti black mold infection.	Masmoudi et al., 2021
*Curtobacterium, Paenibacillus, Pantoea, Sanguibacter and Saccharibacillus*	*Triticum aestivum* L.		Resist fungal infections and reduce the use of pesticides.	Bziuk et al., 2021
*Streptomyces hygroscopicus* OsiSh-2	*Oryza sativa* L.	Expression of related proteins and chloroplasts.	Control the infection of pathogenic bacteria, regulate the plant defense system, and optimize the growth and development of rice.	Gao et al., 2021
*Pantoea, Enterobacter, Pseudomonas, Achromobacter, Xanthomonas, Rathayibacter, Agrobacterium, Pseudoxanthomonas*, and *Beijerinckia*	*Calendula officinalis* L.	Hydrolase and IAA.	Antifungal activity, resist the invasion of pathogenic microorganisms.	Shurigin et al., 2021
*Clonostachys rosea*	*Blumea balsamifera*	Antibiotics.	Resist the invasion of pathogens.	Shu et al., 2020; Kurokawa et al., 2021
*Trichoderma afroharzianum*	*Ficus elastica*	Antibiotics.	Resist the invasion of pathogens.	Ding et al., 2020
*Alternaria alternata, Bacillus amyloliquefaciens, Pseudomonas fluorescens*	*Withania somnifera*	SA, JA, ROS	Enhanced the expression of salicylic acid- and jasmonic acid-responsive genes in the stressed plants.	Mishra et al., 2018a
*Streptomyces* spp. viz.	*Cicer arietinum Linn.*	The contents of phenylalanine ammonia lyase (PAL), polyphenol oxidase (PPO), total phenol and total flavonoids increased	Resistance to pathogen stress, enhance the survival ability of the host, reduce the degree of lipid peroxidation.	Singh and Gaur, 2017
*Streptomyces fradiae, Streptomyces olivochromogenes, Streptomyces collinus, Streptomyces ossamyceticus and Streptomyces griseus*	*Cicer arietinum Linn.*	chitinase	Antifungal (*Sclerotium rolfsii*)	Singh and Gaur, 2016
*Bacillus tequilensis* (PBE1)	*Lycopersicon esculentum*	The contents of IAA, hydroxymate type siderophore increased	Antifungal	Bhattacharya et al., 2019
*Bacillus subtilis*	*Glycine max (Linn.) Merr.*	cell wall degrading enzymes, IAA, etc.	Antifungal (*Macrophomina phaseolina*)	Chauhan et al., 2022

### Endophytes Promote the Production of Host Secondary Metabolites

The production of active substances in endophytes seems to be inseparable from the role of endophytes. Recent studies have shown that when endophytes promote the production of host secondary metabolites, their hosts do not simply increase substances but the endophytes trigger a series of biochemical processes in their hosts, such as host growth and stress resistance regulation. There are two main ways for endophytes to promote the host. One way is that endophytes generate the same signal pathway as their hosts by during gene mutation or information exchange and then produce secondary metabolites similar to their hosts. For example, endophytes isolated from marigold can produce the same hydrolytic enzymes and IAA as their host (Shurigin et al., 2021). Another way is that endophytes cooperate with their hosts to complete a process of the same signal pathway, which may produce key enzymes in the pathway, or change a reaction direction in the process of host metabolism to make their hosts produce specific metabolites (Khattab and Farag, 2021).

## Applications and Research Prospects of Endophytes and Secondary Metabolites

As reliable companions of hosts, endophytes play an important role in the growth and development of hosts and the accumulation of secondary metabolites (Singh et al., 2022). In recent years, with the rapid increase in endophyte-related research, endophytes have gradually become the focus of attention. The study of abundant endophytic species and their response genes suggests their important role in symbiosis systems and mechanisms (Tariq et al., 2022). Studies have shown that under the interaction between hosts and endophytes, hosts can use their own defense system to “screen” specific microorganisms to form a symbiotic system. Conversely, successfully “recruited” endophytes can also make their hosts better adapted to the environment during growth (Wu et al., 2021). Therefore, endophytes are not only natural drug substitutes for promoting host growth and development but also seed banks of new active metabolites. However, due to the complexity of the interactions between species, there are still many unknown fields in research on the lifestyles and action mechanisms of endophytes.

Currently, Endophytic effects have been studied in only a small number of plant species, the vast majority of which come from land plants (Nasiruddin et al., 2020). Studies based on metabolomics have also shown that endophytes are repositories of bioactive metabolites that can produce many active products with pharmacological effects, such as antimicrobial, antitumor, antibiotic, antioxidant, and immune agents (Gupta et al., 2020; Table 4). Although endophytes can continuously and effectively produce several bioactive compounds, it is not realistic to obtain these active substances only from endophytes. Further studies have shown that the acquisition of beneficial active products from endophytes is affected by many internal and external factors, such as the living state of the host plants, species, geographic location, climatic conditions and even the season of sample collection (Li et al., 2020). In fact, the active products of endophytes can solve the shortage of natural resources and provide new age ideas for the development and preparation of new drugs, but current research on endophytes is far from reaching this goal (Yan et al., 2019). At present, although some endophytic strains that can produce host active metabolites have been isolated, almost none of them can be used in production due to the difficulties of *in vitro* culture. Therefore, the problem of *in vitro* propagation of endophytes from hosts and mass production of active ingredients is a key problem faced by current applied research, which is also the premise of endophytes replacing medicinal plants to achieve commercial production of good pharmacodynamically active ingredients (Shao et al., 2021).

**TABLE 4:** Endophyte induced secondary metabolites and their biological activities during plant–endophyte interaction.

Microbial classification	Endophytic strains	Sources	Secondary metabolite instead of product	Functions	References
Endophytic actinomycetes	*Streptomyces* sp.	*Allium tuberosum*	6-prenylindole	Antifungal activity, antitumor.	Singh and Dubey, 2018
	*Streptomyces* sp.	*Bruguiera gymnorrhiza*	Sedecamycin	Anti-HIV activity.	Ding et al., 2010
	*Streptomyces* sp. YINM00001	*Peperomia dindygulensis Miq.*	Antimicrobial and/or anticancer compounds cycloheximide, dinactin, anthracimycin	Antibacterial, anti-tumor.	Liu et al., 2022
Endophytic fungi	*Pyricularia oryza*	*Oryza sativa* L.	Melanin	Antifungal activity.	Motoyama, 2020
	*Alternaria* sp.,*Metarhizium anisopliae, Mucor rouxianus, Pestalotiopsis quepinii, Aspergillus fumigatus*	*Taxus brevifolia*	Paclitaxel	Anticancer activity.	Naik et al., 2019
	*Entrophospora infrequens*	*Nothapodytes foetida*	Camptothecin	Antifungal and cytotoxic.	Munir et al., 2020
	*Paenibacillus*	—	Huperzine	Cholinesterase inhibitors	Kuźniar et al., 2020
	*Nigrospora* sp.,*Chaetomium globosum*	—	Chaetoglobosin A	Activity against root-knot nematodes.	Chowdhary and Sharma, 2017
	*Eupenicillium parvum*	*Azadirachta indica*	Nimbin	Anti-feedant	Kusari et al., 2012b
Endophytic bacteria	*Pseudomonas, Xanthomonas, Variovorax, Bacillus, Inquilinus, Pantoea*, and *Stenotrophomonas*	*Alkanna tinctoria*	Alkannin and shikonin (A/S)	Antibacterial, anti-tumor, promote wound healing, plant growth.	Rat et al., 2021a
	*P. aeruginosa* CP043328.1	*Anredera cordifolia* CIX1	Diisooctyl phthalate and oxadiazole, 5-benzyl-3	Antibacterial and antioxidant activities.	Nxumalo et al., 2020
	*Acinetobacter baumannii*	*Capsicum annuum* L.	Phenol, 2,4-bis(1,1-dimethylethyl)- and phenol, 3,5-bis(1,1-dimethylethyl)-	Antioxidant.	Monowar et al., 2019
	*Bacillus atrophaeus*	*Licorice*	1,2-benzenedicarboxylic acid, bis (2-methylpropyl) ester; 9,12-octadecadienoic acid (Z,Z)-, methyl ester; 9-octadecenoic acid, methyl ester, (E)-; and decanedioic acid, bis(2-ethylhexyl) ester	Antibacterial activity.	Mohamad et al., 2018
	*Microbacterium* sp.	*Catharanthus roseus*	Vindoline	Hodgkin’s disease and acute leukemia.	Anjum and Chandra, 2019
	*Pseudomonas fluorescens*	*Atractylodes lancea*	IAA	Promote root development and carbohydrate uptake.	Zhou et al., 2018
	*Microbacterium*, *Burkholderia*	*Coptis teeta*	Berberine	Anti - inflammatory, anti - tumor, reduce blood sugar activity.	Liu et al., 2020
	*Bacillus subtilis*	*Ligusticum chuanxiong*	Ligustrazine	Treatment of ischemic vascular diseases.	Yin et al., 2019
	*Bacillus velezensis* Bvel 1	*Vitis vinifera* L.	Iturin A2, surfactin-C13 and -C15, oxydifficidin, bacillibactin, L-dihydroanticapsin, and azelaic acid	Antifungal activity, promote plant wound healing.	Nifakos et al., 2021

Overall, the active metabolites produced from the interactions between endophytes and host plants have great potential in future, and there is a large demand in the fields of medicine, agriculture, biodegradation and bioremediation. In recent years, with the improvement and application of HPCE, HPLC–MS and other technologies, the rapid identification of active metabolites of plant endophytes has become possible, and endophyte based nanoparticles are expected to play an important role in drug development in future (Pentimone et al., 2020). For instance, Zhang D. et al. (2021) isolated and identified a new endophytic bacterium, *Bacillus altitudinis* SB001, from wild sweet grass in China. Transcriptome sequencing showed that mature enzyme K, Tetratricopeptide repeats (TPR)-like superfamily proteins, Lateral organ boundaries (*LOB*) domain proteins and Broad-complex (*BTB*)/pox virus (*POZ*)/PDZ-binding motif (*TAZ*) domain proteins may play a role in the growth promotion of wild Chinese sweet grass (Zhang D. et al., 2021). In addition, the main promoters in the interactions with their host, the MFS transporter and DNA rotase subunit B, were also found in *Bacillus altitudinis* SB001. These findings suggest that endophytes may be useful candidates for host growth promotion.

## Perspectives

Endophytes are a kind of microbial resource with abundant species and wide host. Most endophytes can regulate the growth, development and metabolism of their host. Therefore, a comprehensive and in-depth study of endophytes is of great significance. At present, research on endophytes is still in an early stage of relative development, and the embodiment of their application value still needs in-depth research and improvement. Although the whole genome of some endophytes has been deciphered, there are still many aspects to be clarified in endophyte research, especially the symbiotic mechanisms between endophytes and hosts, which remain to be further explored. Only by deeply understanding the interactive mechanisms between endophytes and their hosts can we further explore the potential value of endophytes in improving the growth and development of their hosts and the production of active metabolites. Currently, research on endophytes faces four major problems. In terms of endophyte invasion of their hosts, their mode of action still needs to be discussed, such as invasion site and invasion form (spores, hyphae, etc.). The study of the mechanisms of interactions between endophytes and their hosts, such as the specific ways of endophyte colonization, whether the endophyte can achieve proliferation after colonization and whether their host has antagonistic reactions, is still unclear. In terms of host growth and development, how endophytes regulate host metabolism, such as producing new metabolites and “reprocessing” host metabolites, still needs to be further revealed. In terms of production and application, problems such as difficulty in breeding on a large scale *in vitro* and the sharp decline of the effects *in vitro* are still serious.

Solving the above problems will not only help to tap into the ecological functions of endophytes but will also further improve the application potential of endophytes and will provide a base for the further development and utilization of endophytes. At present, although there have been an increasing number of studies on endophytes, there have been few reports on the large-scale application of endophytic preparations and their active metabolites in commercial production. The utilization of endophyte biological resources is still difficult, and there is no effective detection technology to directly identify endophytic bacteria *in vivo* from their host. *In vitro* endophyte isolation, culture and even fermentation cannot accurately obtain the corresponding strains or even metabolites. In addition, there are interactions between endophytes in the host, which undoubtedly adds difficulties in endophytes study. The mystery of endophytes is gradually being revealed. In future, on the basis of ensuring the biological activity of isolated and cultured endophytes, the improvement of their characteristics and application in basic research and commercial production will be of great significance. For example, new drugs can be created to treat diseases and for agricultural production. Regulating host gene expression and pathways improves the growth of valuable medicinal plants, realizing transgenic breeding and improving crop quality. Now is the time to elevate endophyte research from traditional physiological and biochemical research to higher cellular- and molecular-level research. Combined with omics technology, a database of endophytes and their active metabolites should be established. Then, the database should be used to understand the unknown field of endophytes and host interactions and to benefit from it.

## Author Contributions

DX and LL conceived, supervised, and wrote and reviewed the manuscript. ZL, WW, YH, and MQ originally wrote and reviewed the draft. LL, DX, and YH co-founded and co-administrated the project. All authors read and approved the final version.

## Conflict of Interest

The authors declare that the research was conducted in the absence of any commercial or financial relationships that could be construed as a potential conflict of interest.

## Publisher’s Note

All claims expressed in this article are solely those of the authors and do not necessarily represent those of their affiliated organizations, or those of the publisher, the editors and the reviewers. Any product that may be evaluated in this article, or claim that may be made by its manufacturer, is not guaranteed or endorsed by the publisher.
